# Influence of gamma radiation and phenylalanine on secondary metabolites in callus cultures of milk thistle (*Silybum marianum* L.)

**DOI:** 10.1186/s43141-022-00424-2

**Published:** 2022-12-15

**Authors:** Asmaa M. Khalifa, Eman Abd-ElShafy, Rasha Abu-Khudir, Reda M. Gaafar

**Affiliations:** 1 Botany and Microbiology Department, Faculty of Science, Al Azhar University (Girls Branch), Cairo, Egypt; 2 Chemistry Department, College of Science, King Faisal University, Al-Hofuf, Al-Ahsa, 31982 Saudi Arabia; 3 Chemistry Department, Biochemistry Branch, Faculty of Science, Tanta University, P.O. Box 31527, Tanta, Egypt; 4 Botany Department, Faculty of Science, Tanta University, P.O. Box 31527, Tanta, Egypt

**Keywords:** Milk thistle, In vitro, Precursors, Flavonoids, Phenols

## Abstract

**Background:**

A useful technique for growing large amounts of plant material is in vitro propagation of important medicinal plants. The present investigation deals with the enhancement of secondary metabolite production via elicitation using gamma (γ)-radiation and phenylalanine (Phe) precursor feeding in callus cultures of *Silybum marianum* L.

**Results:**

Seeds were exposed to two doses of γ-radiation (25 and 50 Gy) and the calli derived from stem explants  obtained from seedlings of these radiated seeds were treated with different concentrations of Phe. The biosynthesis of phenols and flavonoids was evaluated. It was found that callus cultures derived from explants of the seeds exposed to 25 Gy γ-radiation and treated with 4 mg/l Phe accumulated the maximum phenolic content (34.27±0.02 mg/g d.wt.), while the highest flavonoid content (9.56±0.12 mg/g d.wt.) was found in callus cultures derived from explants of seeds radiated with 25 Gy γ-radiation and subjected to 1 mg/l Phe. Similarly, HPLC quantification revealed that the production of flavonoids was highly accumulated (1343.06 μg/mg d.wt.) in callus cultures from explants of seeds  exposed to 25 Gy γ-radiation and grown at 1 mg/l Phe compared to the other treatments. In addition, a total of 11 important flavonoids have been determined in all callus cultures, except for acacetin-7-O-rutinoside, which was not found in the callus culture of the control.

**Conclusions:**

These findings suggest that γ-radiation combined with Phe can improve the metabolism of *S. marianum* L. and could be used to produce such valuable metabolites on a commercial scale.

## Background

 Milk thistle, *Silybum marianum* (L.) Gaertn., is among the most ancient of all known herbal medicines. Various preparations of the plant, especially the fruits, have been used medicinally for over 2000 years, mainly for the treatment of liver disorders [1]. Silymarin is a constitutive natural compound that accumulates in the fruits of *S. marianum* L.  is composed of an isomeric mixture of the flavonolignans silychristin, isosilychristin, silydianin, silybin A, silybin B, isosilybin A, and isosilybin B. It is considered to be the principle pharmacologically active constituent in *S. marianum* L.  [1–3]. The traditional cultivation of *S. marianum* L. plants has several limitations that cause a reduction in the total yield of silymarin. Plants of *S. marianum* L. are difficult to manually manipulate, particularly during harvesting, due to the spiny margins of leaves and flowers [4]. Thus, a possible alternative method for the large-scale production of silymarin is to use plant cell and organ cultures, where the yield depends on the constituents of the culture medium [5]. However, without elicitation, the yield of these compounds is poor [6]. Among different culture systems, callus culture may be considered an important initial biotechnological step for the large-scale production of biomass and a starting material for cell suspension cultures and shoot regeneration, being exploited for the accumulation of active compounds [7].

In vitro culture of important medicinal plants has become a reliable technique for high amounts of plant material production [8]. Production of secondary metabolites *via* plant cell cultures yields various advantages, including the extensive manipulation of the biosynthesis of bioactive compounds using large quantities of vegetal material under sterile and controlled conditions [9, 10] as well as higher productivity without seasonal harvesting and potential cross-contamination compared to field-grown plants [11]. Flavonoids are a class of secondary metabolites with the basic structure of two aromatic rings (A and B) linked by a heterocyclic pyrane ring (C).  They are water-soluble phenolic glycosides that impart color to flowers and fruits of higher plants. They have also been linked to a reduction in the risk of cardiovascular diseases and post-climacteric osteoporosis [12]. Among these, quercetin is regarded as an active component with a variety of biological effects, such as anti-inflammatory, anti-cancer, antibacterial, antiviral, anti-gonadotropic, and anti-hepatotoxic activities [13]. In order to enhance the synthesis of secondary metabolites, several organic compounds have been fortified in the culture medium [14]. The rationale behind this approach is the good possibility that any substance that is an intermediate in or at the start of a secondary metabolite biosynthetic route would increase the yield of the final product [15]. Supplementing precursors, elicitors, and growth promoters have also been shown to increase secondary metabolite content in cell cultures [16].

Electromagnetic radiation, including gamma (γ)-rays, X-rays, visible light, and ultraviolet, is known to influence plant growth and development by inducing morphological, structural, and functional changes in cells and tissues [15]. γ-irradiation is sparsely ionizing electromagnetic radiation that can directly or indirectly affect the growth and physiology of plants. The direct effects of γ-irradiation on plants include induction of DNA mutation and chromosomal aberrations, while the increase in reactive oxygen species (ROS) and oxidative stress (OS) are considered as their indirect effects [17, 18]. For several years, γ-irradiation has been regarded as a new rapid method to change the qualitative and quantitative characteristics of plants [19, 20]. Low-dose ionizing irradiation affects cell growth, proliferation, germination rate, enzyme activity, as well as stress resistance [21]. The γ-irradiation technique is one of the most important methods used to create mutants with desired traits of agricultural significance [22]. γ-rays are highly reactive ionizing radiation that interacts with the atoms and molecules of exposed plant materials to produce free radicals, which are actively participating in various processes within the cells [23].

Flavonoids are the most ubiquitous group of natural polyphenols known for their photoprotective and antioxidative roles in the stress acclimation of plants, especially as UV filters [24]. In comparison to flavonoids generated from intact plant tissues, those derived from plant cell cultures are easier to separate in the polymeric form [25–27]. By regulating the in vitro conditions and feeding precursors to induce metabolite synthesis, their concentrations were markedly increased in cell cultures [28]. Phenylalanine (Phe), an upstream metabolic precursor via the phenylpropanoid pathway, is the source of flavonoids. Supplementation of Phe is expected to increase the level of the target compound [29].

Feeding culture media with phenylalanine as a precursor of the phenylpropanoid biosynthetic pathway resulted in a three-to fivefold increase in 5-methoxypodophyllotoxin in suspension culture of *Linum flavum* [30]. Phenylalanine enhanced the production of Taxol in suspension cell cultures of *Taxus baccata* [31]. In tissue cultures of *Hydrocotyle bonariensis* [25] and *Silybum marianum* L. [16, 32], the impact of Phe on flavonoids has been investigated. It has been found that 20 Gy γ-irradiation increased total phenolic and flavonoid accumulation in rosemary callus culture [17]. In addition, the dose of 10 Gy γ-irradiation increased the phenolic acid content in cinnamon [33]. Likewise, Masoud et al. [34] stated that a dose of 40 Gy increased the accumulation of total phenolic compounds in *Cichorium pumilum* Jacq. roots, while at 600 Gy the highest phenol content of *Abelmoschus moschatus* was produced [35]. The importance of phenols for plants is due to a fair correlation between antioxidant/free-radical scavenging activity and its phenolic content. Furthermore, phenol compounds may protect plants from irradiation-induced OS [34]. The main objective of this work  was to enhance the production of natural medical components in *S. marianum* L. callus cultures using phenylalanine and γ-irradiation as elicitors.

## Methods

### Plant material and callus induction

The seeds of *S. marianum* L. were collected from its wild habitats, from Gharbia governorate at wheat field edges. The collected seeds were then treated by two doses of γ-irradiation (25 and 50 Gy). Exposure to γ-rays was carried out at the Egyptian Atomic Energy Authority with Mega Gamma-1 type J 6600 cobalt-60 as the irradiation source. The seeds (untreated, exposed to 25 Gy and 50 Gy) were washed under running tap water. They were surface sterilized by dipping in 70% ethanol for 1 min, immersed in 50% Clorox solution containing 5.4% sodium hypochlorite (NaOCl) with one drop of a commercial detergent for 20 min, and rinsed with sterilized distilled water for five times. Surface sterilization of seeds was carried out under complete aseptic conditions in the Laminar Air Flow Hood to prevent bacterial or fungal contamination of the explants. The sterilized seeds were germinated aseptically on half-strength Murashige and Skoog (MS) medium [36], supplemented with 30 g/l sucrose and 10 g/l agar. Cultures were incubated at 25 ± 2°C in the dark to facilitate germination. The clean and sterilized stem explants obtained from germinated seeds were cut into 1 cm segments and inoculated on solid MS medium supplemented with a different concentration of 2,4-dichlorophenoxy acetic acid (2,4-D) alone or in combination with 6-benzyladenine (BA) or kinetin (Kn) to induce callus formation. After 7 weeks, the successfully induced callus was separated from the explants and cultured separately until a sufficient amount of callus was produced.

### Culture medium and conditions

The callus (derived from the stem explants) obtained from the three treatments was grown on MS medium supplemented with the best callus induction plant growth regulators (PGRs) combination (1 mg/l 2,4-D and 0.5 mg/l BA), treated with phenylalanine at various concentrations (1, 2, 3, and 4 mg/l) and incubated at 25 ± 2°C in the dark. After 7 weeks, the calli were separated from the medium and dried in the oven at 40°C for 12 h.

### Estimation of total phenols

The total phenolic content of *S. marianum* L. calli  was estimated quantitatively using the method described by Jindal and Singh [37]. In brief, 1 ml of the ethanolic extract was mixed with 0.1 ml of Folin reagent and 1 ml of 20% Na_2_CO_3_, then completed up to a known volume (5 ml) with distilled water. Thereafter, the absorbance was measured using the UV spectrophotometer, JENWAY (Japan) at 650 nm after 30 min. A standard curve was plotted using different concentrations of gallic acid (GA) for the determination of the total phenolic content (mg/g d.wt).

### Determination of total flavonoids

The total flavonoids content of *S. marianum* L. calli  was extracted by soaking a known weight (0.1 g) of the dried callus in 10 ml of 95% ethanol in a water bath at 60°C for 4 h. The clear supernatants were diluted to a known volume (10 ml). The aluminum chloride colorimetric method was used for total flavonoids estimation [38]. 0.5 ml of callus ethanolic extract was separately mixed with 1.5 ml of 95% ethanol, 0.1 ml of 10% aluminum chloride, 0.1 ml of 1 M potassium acetate, and 2.8 ml of distilled water. Then, it was incubated at room temperature for 30 min, after which the absorbance of the reaction mixture was measured at 415 nm  using the UV spectrophotometer, JENWAY (Japan). The calibration curve was plotted using quercetin as a standard flavonoid. The amount of 10% aluminum chloride was replaced by the same amount of distilled water as the blank. Similarly, 0.5 ml of methanol extract of quercetin was used as a standard. The concentration of total flavonoids was expressed as mg/g d.wt.

### Quantification analysis of flavonoids

High performance liquid chromatography (HPLC) was used for the qualitative and quantitative analysis of flavonoids in the calli  of *S. marianum* L. The calli (from different treatments) were oven-dried and 0.1 g of the sample was used for extraction with methanol at 80°C for 4 h. The extracts were dried and re-dissolved in 1 ml of methanol [39]. The HPLC system of Shimadzu LC-10Avp plus with a PDA detector (SPD-M20A) and a C18 column (4.6 mm × 250 mm, 5 μm, Eclipse XDB C18) was used for the qualitative and quantitative analysis of total flavonoids in the methanol extracts. The mobile phase contained acetonitrile (A) and 0.1% trifluoroacetic acid (B). A gradient method was used for the separation of the extracted samples: 0–25 min, 15–40% A; 25– 40 min, 40–100% A. The elution flow rate and detection wavelength were set at 1.0 ml/min and 254 nm, respectively. The identity of peaks separated by HPLC was confirmed by the injection of flavonoid standards obtained from Sigma Aldrich, and UV spectral analysis was carried out to confirm the identification of flavonoids. The HPLC analysis was conducted in the microanalysis laboratory of the Food Technology Research Institute, Agriculture Research Center, Giza, Egypt.

### Statistical analysis

The experiments were independently repeated three times under the same conditions, and the concentrations and all analyses were performed in triplicate. Results are expressed as mg/g d.wt. for flavonoids and phenols accumulated in the treated callus cultures compared to untreated calli. The graphs depicting the total flavonoids accumulation were created using Microsoft® Excel. Data were statistically analyzed using one-way ANOVA followed by Duncan’s new multiple range tests as described by Snedecor and Cohchran [40]. Means followed by the same letters are not significantly different at *P* ≤ 0.05.

## Results

### Callus induction

**Fig. 1:**
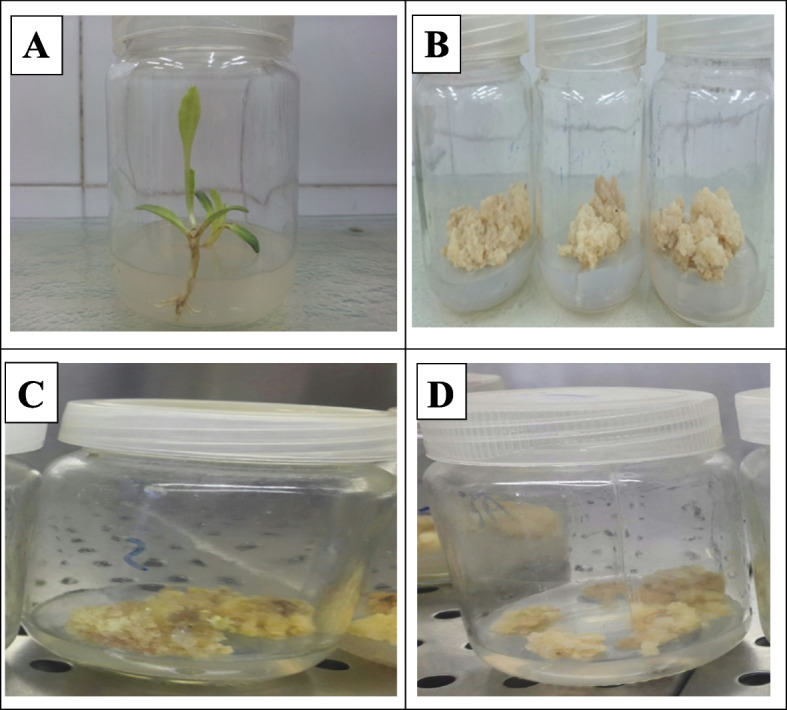
Seed germination of *S. marianum* L. on half-strength Murashige and Skoog (MS) medium (**A**), calli  formation from stem segments on MS medium supplemented with 1 mg/l 2,4-D and 0.5 mg/l BA (**B**), and calli  from stem explants derived from seeds exposed to 25 Gy (**C**) or 50 Gy (**D**)

The seeds cultured on half strength basal MS (devoid of any PGRs) germinated normally (Fig. 1A) and developed into seedlings with roots and shoots without callus formation. Stem explants derived from 14-days-old seedlings were cultured on MS medium with different concentrations and combinations of 2.4-D and BA or Kn for callus induction (Table 1). The optimum callus induction (about 95%) was obtained at 1 mg/l 2.4-D and 0.5 mg/l BA and the first sign of callus growth from the explants was noticeable between 15-20 days after subculturing in the fresh medium. Callus induction was initiated above the surface of the explant and was friable and granulated (Fig. 1B).

**Table 1:** Effect of different concentrations of plant growth regulators (PGRs) on callus induction from stem explants of *S. marianum* L.

PGRs (mg/l)			Callus induction (%)	Callus color	Callus texture
2,4-D	BA	Kn			
1.0	0.0	0.5	60^c^	Light brown	Compact
1.0	0.0	1.0	60^c^	Light brown	Compact
1.0	0.0	1.5	50^d^	Light brown	Compact
1.0	0.0	2.0	50^d^	Light brown	Compact
1.0	0.5	0.0	95^a^	Creamy white	Friable and granulated
1.0	1.0	0.0	90^a^	Creamy white	Friable and granulated
1.0	1.5	0.0	88^b^	Creamy white	Friable and granulated
1.0	2.0	0.0	85^b^	Creamy white	Friable and granulated
0.5	0.0	0.0	55^d^	Whitish yellow	Friable and granulated
1.0	0.0	0.0	65^c^	Whitish yellow	Friable and granulated
1.5	0.0	0.0	60^c^	Whitish yellow	Friable and granulated
2.0	0.0	0.0	60^c^	Whitish yellow	Friable and granulated

Different letters indicate a significant difference at *P* ≤ 0.05

### Combined effects of γ-irradiation and phenylalanine (Phe) on the accumulation of total phenols and flavonoids

#### Total phenols and flavonoids

**Fig. 2:**
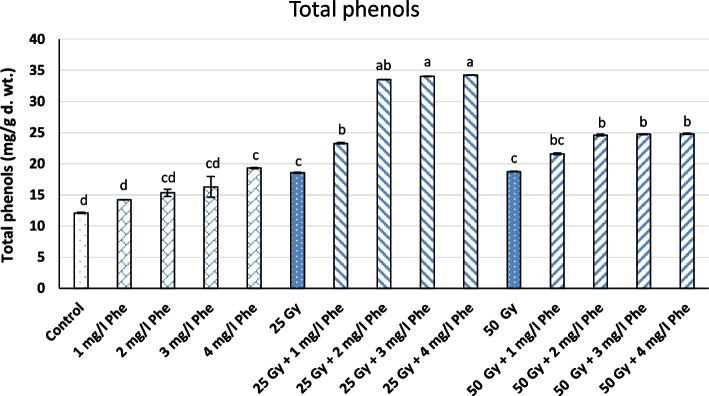
Total phenolic content under treatment of different combinations of γ-radiation (25 and 50 Gy) and Phe (1, 2, 3, and 4 mg/l) in the callus cultures of *S. marianum* L. Different letters on the bars indicate a significant difference at *P* ≤ 0.05

The combinations of γ-radiation (25 and 50 Gy) and different Phe concentrations were found to have astonishing effects on the biosynthesis of phenolic compounds in the callus culture of *S. marianum *L., where the callus cultures treated with different combinations showed higher levels of phenols and flavonoids than those of the controls.  The results indicated that the highest accumulation of total flavonoids of 9.56±0.12 mg/g d.wt. was found in callus cultures subjected to 25 Gy γradiation and 1 mg/l Phe (Fig. 2 and Table 2). This was followed by callus cultures subjected to 50 Gy γ-radiation and 1 mg/l Phe (8.82±0.07 mg/g d.wt.). Likewise, the highest level of total phenolic content (34.27±0.02 mg/g d.wt.) was found in callus cultures subjected to 50 Gy γ-radiation and 4 mg/l Phe in comparison to control (12.10± mg/g d.wt.), which was significantly lower. When applied alone, it was noticed that the content of flavonoids increased more with a 25 Gy dose of γ-radiation (8.08±0.10 mg/g d.wt.) than with a 50 Gy dose (7.43±0.06 mg/g d.wt.). However, both doses (25 and 50 Gy) showed higher phenolic content (18.57 and 18.76 mg/g d.wt., respectively) compared to control (Fig. 2 and Table 2).

**Table 2:** Effect of different combinations of γ-radiation (25 and 50 Gy) and Phe (1, 2, 3, and 4 mg/l) on the total phenolic and flavonoid content in the callus cultures of *S. marianum* L.

Treatments	Total phenols (mg/g d.wt.)	Total flavonoids (mg/g d.wt.)
Control	12.10±0.10^d^	8.17±0.03^bc^
Free +1 mg/l Phe	14.21±0.01^d^	8.61±0.08^b^
Free +2 mg/l Phe	15.01±0.59^cd^	8.73±0.15^b^
Free +3 mg/l Phe	15.33±1.67^cd^	8.01±0.09^c^
Free +4 mg/l Phe	19.39±0.10^c^	7.77±0.14^c^
25 Gy	18.62±0.07^c^	8.08±0.10^bc^
25 Gy + 1 mg/l Phe	23.37±0.12^b^	9.56±0.12^a^
25 Gy + 2 mg/l Phe	33.56±0.02^ab^	8.41±0.09^bc^
25 Gy + 3 mg/l Phe	34.06±0.03^a^	8.46±0.04^bc^
25 Gy + 4 mg/l Phe	34.27±0.02^a^	7.82±0.08^c^
50 Gy	18.80±0.07^c^	7.43±0.06^d^
50 Gy + 1 mg/l Phe	21.63±0.15^bc^	8.82±0.07^b^
50 Gy + 2 mg/l Phe	24.56±0.14^b^	8.60±0.11^b^
50 Gy + 3 mg/l Phe	24.81±0.07^b^	8.16±0.12^bc^
50 Gy + 4 mg/l Phe	24.94±0.10^b^	8.01±0.01^bc^

Different letters indicate a significant difference at *P* ≤ 0.05

#### HPLC analysis of total flavonoids

**Fig. 3:**
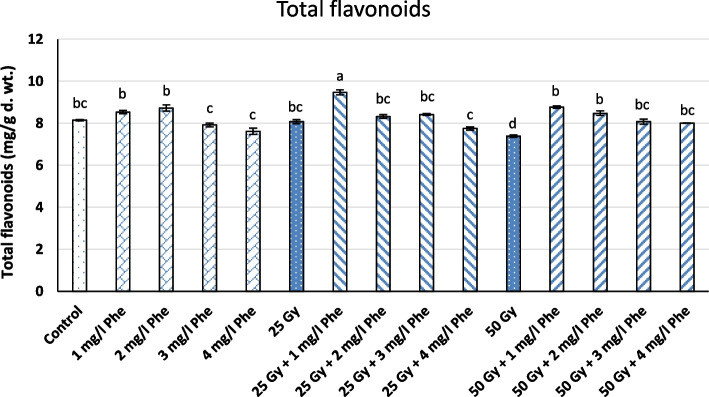
Total flavonoid content under treatment of diffrent combinations of γ-radiation (25 and 50 Gy) and Phe (1, 2, 3, and 4 mg/l) in the callus cultures of *S. marianum* L. Different letters on the bars indicate a significant difference at *P *≤ 0.05

**Fig. 4:**
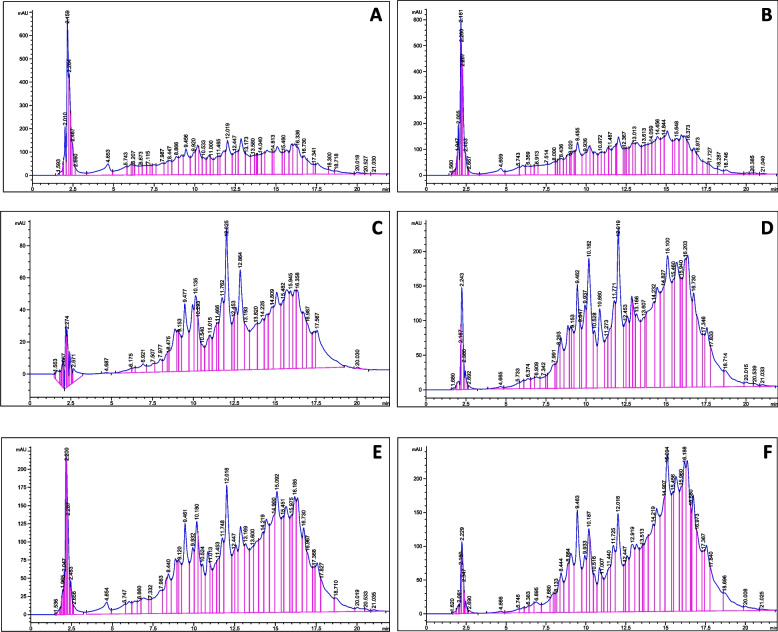
Chromatograms of total flavonoids determined in callus cultures of *S. marianum* L. treated with different combinations of γ-radiation (25 and 50 Gy) and the amino acid precursor Phe (1 mg/l). **A**: control, **B**: 1 mg/l Phe, **C**: 25 Gy γ, **D**: 25 Gy γ + 1 mg/l Phe, **E**: 50 Gy γ, **F**: 50 Gy γ + 1 mg/l Phe

A total of 11 important flavonoids have been determined  in callus cultures of *S. marianum* L. treated with a combination of γ-irradiation (25 and 50 Gy) and 1 mg/l Phe using HPLC (Table 3 and Fig. 3). The raised aggregation of these compounds was noticed in cultures stressed with γ-radiation and Phe compared to controls (Table 3 and Fig. 4). HPLC evaluation showed that the highest accumulation of total flavonoids (1343.06 μg/mg d.wt.) occurred in callus cultures exposed to the dose of (25 Gy) and 1 ml of Phe. In contrast, the least production (763.56 μg/mg d.wt.) was found in callus cultures derived from explants of seeds exposed to 50 Gy (Table 3). Furthermore, the highest levels of kaempferol biosynthesis (37.53±0.35 and 37.63±0.38 μg/mg d.wt.) were observed in callus cultures from explants derived from seeds treated with γ-radiation at a dose of 25 and 50 Gy, respectively. Likewise, the maximum accumulation of quercetin (384.53±6.58 μg/mg d.wt.) and rutin (62.92±0.11 μg/mg d.wt.) were observed with the treatment 25 Gy and 1 mg/l Phe, whereas the highest level of apigenin (47.15±0.78 μg/mg d.wt.) and kaempferol-3-(2"-p-comaroyl) glucoside (469.30±1.02 μg/mg d.wt.) were found in the callus cultures fortified with 1 mg/l of Phe alone. Furthermore, the maximum level of quercitrin (46.43±0.32 μg/mg d.wt.) was found in callus cultures derived from stem explants from seeds exposed to γ-radiation at a dose of 25 Gy (Fig. 4).

**Table 3:** Total flavonoids determined by HPLC in callus cultures of *S. marianum* L. treated with different combinations of γ-radiation (25 and 50 Gy) and the amino acid precursor Phe (1 mg/l)

Treatment	Total flavonoids (μg/mg d.wt.)										
	Rutin	Naringin	Rosmarinic acid	Quercitrin	Apigenin-7-glucoside	Quercetin	Naringenin	Kaempferol-3-(2"-p-comaroyl) glucoside	Kaempferol	Acacetin-7-O-rutinoside	Apigenin
Control	62.58^a^ ±0.39	24.47^d^ ±0.15	4.23^e^ ±0.07	3.17^e^ ±0.03	38.80^c^ ±0.20	258.33^c^ ±0.99	65.47^c^ ±0.74	407.24^b^ ±1.83	11.83^c^ ±0.08	-	17.30^c^ ±0.51
1 mg/l Phe	48.72^b^±0.58	60.40^c^ ±0.82	13.12^a^ ±0.08	6.90^d^ ±0.05	57.32^b^ ±0.65	257.97^c^ ±1.53	98.30^a^ ±1.11	469.30^a^ ±1.02	21.27^b^ ±0.25	48.13^d^ ±0.85	47.15^a^ ±0.78
(25 Gy)	26.85^d^±0.40	64.47^c^ ±0.29	9.33^d^ ±0.15	46.43^a^ ±0.32	77.80^a^ ±0.70	256.38^c^ ±2.75	45.67^d^ ±0.49	468.99^a^ ±1.86	37.53^a^ ±0.35	87.47^c^ ±0.38	28.84^b^ ±0.35
(25 Gy)+ 1 mg/l Phe	62.92^a^±0.11	96.70^a^ ±0.36	12.07^b^ ±0.04	35.50^b^ ±0.26	77.63^a^ ±0.15	384.53^a^ ±6.58	75.40^b^ ±1.05	445.62^ab^ ±0.29	11.43^c^ ±0.31	100.34^b^ ±0.85	23.05^b^ ±0.41
(50 Gy)	49.44^b^ ±0.83	88.43^b^ ±0.42	7.49^c^ ±0.09	32.57^b^ ±0.21	24.37^d^ ±0.38	255.04^c^ ±1.02	44.70^d^ ±0.78	234.55^c^ ±3.35	37.63^a^ ±0.38	13.97^e^ ±0.21	8.83^d^ ±0.16
(50 Gy)+1 mg/l Phe	44.89^b^c±0.60	80.83^bc^ ±0.21	13.36^a^ ±0.12	18.73^c^ ±0.15	27.50^d^ ±0.53	270.94^b^ ±3.52	56.00^cd^ ±0.70	406.65^b^ ±2.47	9.72^d^ ±0.13	141.33^a^ ±1.85	13.35^cd^ ±.27

Different letters indicate a significant difference at *P* ≤ 0.05

## Discussion

Gamma radiation has been considered a rapid and reliable method for inducing variation in a plant’s metabolic and physiological processes [41]. Hence, γ-irradiation has gained popularity in recent decades as a novel elicitation technique for the enhanced production of secondary metabolites in plant organs and cell cultures [42, 43]. The interaction of the applied γ-rays and the free radicals in plant cells activates signal molecules involved in the defense system and synthesizes secondary metabolites [17, 42, 44, 45]. A higher level of different types of metabolites, including phenolics, terpenoids, and alkaloids, has been reported in many medicinal plants by γ-irradiation [46].

Previous studies using γ-radiation showed that it could increase the amount of secondary metabolites in callus and shoot cultures. This was observed in the callus cultures of *Lithospermum erythrorhizon* [47], *Rosmarinus officinalis* [17], *Panax ginseng* [45], *Nothapodytes foetida* [48], *Stevia rebaudiana* [7], *Sesuvium portulacastrum* [49], *Rubia cordifolia* [43] and shoot cultures of *Mucuna pruriens* [50]. Gamma radiation could increase radical scavenging activity by induction of phenolic compounds. Previously, it has been shown that phenolic content in *Ferula gummosa* was increased under γ-irradiation at 20 and 25 Gy, and the best dose was reported to be 20 Gy according to growth response [51].

The present study showed that γ-irradiation alone or combined with Phe increased the phenolic and flavonoid content in *S. marianum* L. callus cultures. Treatment with 25 and 50 Gy increased phenolic and flavonoid content, where the highest contents were observed at 25 Gy. So, 25 Gy can be used as a tool for secondary metabolite induction in *S. marianum* L. callus cultures. Based on the data in Table 3, it was noticed that increasing the concentration of γ-radiation resulted in a decrease in the total flavonoid content in the callus cultures of *S. marianum* L. The reduced levels of phenolic and flavonoid accumulation occurred mostly at higher doses of exposure. The underlying mechanistic reasons include high level of ROS generation, biological membrane disruption, photoinhibition, impairment of lipid metabolism, DNA damage, and photosystem II destruction [52, 53]. Irradiation exhibited a negative impact on plants, including the direct destruction of plastoquinone (PQ) in chloroplasts, disruption of mitochondrial activities, DNA integrity, the production of reactive oxygen species (ROS), and the formation of peroxyl radicals (ROO^•^) [54].

The results of the current study are in accordance with those of Azeez et al. [42], who concluded that the treatment of callus cultures with 10 Gy γ-radiation dose was effective in stimulating callus biomass production and phytochemical accumulation of *Hypericum triquetrifolium* Turra. However, higher doses led to a reduction of these compounds, especially with regard to irradiation. Similarly, γ-elicitation is considered a potential technique that enhances the production of secondary metabolites such as alkaloids and terpenoids in the callus of different plants, such as *Eurycoma longifolia* [55] and *Vitis* *vinifera* L. [56].

The outcome of the current research was supported by the study of Patil et al. [57], who declared that γ-irradiation at 5 Gy enhanced the growth of *A. annua* callus culture, whereas higher doses significantly reduced calli growth as compared to non-irradiated cultures. Similarly, Le et al. [45] documented that mutant adventitious root cultures of *Panax ginseng* exposed to 20 Gy γ-radiation showed more vigorous growth than the non-irradiated roots. Also, Heydari et al. [58] stated that low doses of γ-radiation favored callus growth in *Salvia nemorosa*, and a significant increase was found in the biomass growth of cultures that were subjected to 10 and 20 Gy doses. In a similar study, El-Garhy et al. [59] concluded that γ-rays (200 and 600 Gy) and colchicine (0.05%) did not inhibit the formation of *S. marianum* L. calli and promoted cell culture growth. Thus, callus induction from leaf segments of seedlings obtained from seeds treated with γ-rays (200 and 600 Gy), 0.05% colchicine, or control conditions, showed high biomass production. The viability of the cell suspension cultures was over 90%. The flavonolignan profile of the radiated seeds confirmed the promising impact of the two studied treatments, given that 200 and 600 Gy positively influenced the total silymarin production.

In their study on the dose-and time-dependent effects of γ-irradiation on germination rate, plant growth, chlorophyll content, oxidative stress (OS), gene expression, and antioxidant activity changes,  Hong et al. [60] reported that long-term exposure to γ-rays had a significantly negative effect on the seed germination percentage. Also, they observed no differences in the final germination percentage between control and irradiated seeds. However, there was a significant difference in the germination pattern between short-term and long-term irradiation in the early germination stages. Germination-related indicators showed a tendency to decrease as the γ-radiation dose and exposure time increased. Mechanistically, the stress raised by UV light activates defense mechanisms in plants along with producing phytoalexin [61]. The production of these plant defensive compounds, including antioxidative enzymes, secondary metabolites, and cell wall modifications, enables them to cope with the oxidative damage prompted by UV radiation through scavenging lethal ROS [62].

In this study, the incorporation of Phe and γ-radiation increased the yield of total phenols and flavonoids in comparison to the control, whereas Phe alone has a low effect (nearly equals to the control). Alongside, recent approaches include combinatorial use of elicitors for more efficient procedure and increased production of biomass. Different light regimes and ultraviolet-C (UV-C) radiation  have been shown to synergistically enhance the production of lignans and neolignas in *Linum usitatissimum* L. in vitro culture. Application of UV-C radiation and melatonin  has been reported to enhance phenolic accumulation and antioxidant activities in callus cultures of *Ocimum* *basilicum* L. [63]. The impact of UV-C radiation and melatonin was also affirmed on the biosynthesis of anti-diabetic phytochemicals and antioxidants in in vitro cultures of *Lepidium sativum* L. by Ullah et al. [64].

Increased total phenols and flavonoids in irradiated plants were also reported by El-Beltagi et al. [17] in rosemary. γ-irradiation can induce the release of phenolic compounds from glycosidic components and degrade the larger phenolic compounds into smaller ones, which finally increases the total phenolic content [65]. Irradiation causes radiolysis of water and helps to produce free radicals such as hydroxyl radical (OH^•^), hydroperoxyl radical (HOO^•^), and hydrated electrons. These radicals may break the glycosidic bonds of procyanidin trimer, tetramer, and hexamer that are present in plants, which increases the total phenolic and flavonoid content in irradiated plants [66]. On the other hand, Abou-Zeid and Abdel-Latif [67] found that the increase in phenylalanine ammonia-lyase (PAL) concentration and activity occurred in *Triticum aestivum* L. seedlings with increasing doses of γ-irradiation. Plant phenylpropanoids or phenolics are synthesized by PAL, a key enzyme in the first step of the phenylpropanoid pathway.

Increased phenolic content under γ-radiation could be related to changes in the enzyme activity of the phenolic biosynthesis pathway [62]. Generally, plants receive various concentrations of radiation that trigger diverse signal transduction pathways. This induces effects on the secondary metabolism of plant species and eventually results in the accumulation of distinct levels of plant metabolites [68].

## Conclusions

The results of this study proved that γ-radiation as an elicitor alone or combined with the precursor amino acid Phe could be used to improve secondary metabolite production from *S. marianum* L. callus cultures, which could be applied on a commercial scale to provide these medicinally important compounds.

## Data Availability

The data used in this study are available upon request through the corresponding author.
